# Post-Ortho-K Corneal Epithelium Changes in Myopic Eyes

**DOI:** 10.1155/2022/3361172

**Published:** 2022-05-29

**Authors:** Dongyi Qu, Yuehua Zhou

**Affiliations:** ^1^Beijing AIER Fukang Eye Hospital, China; ^2^Ming Vision Eye Clinic, Beijing, China; ^3^Ineye Hospital of Chengdu University of TCM, China

## Abstract

The study is aimed at evaluating corneal epithelial thickness changes associated with overnight orthokeratology (ortho-K). In this retrospective study, epithelial thickness was measured using optical coherence tomography (OCT) before and after 1 day, 1 week, 1 month, and 3 months ortho-K nightly lens wear. Compared with pre-orthokeratology measurements, central (2 mm) corneal epithelium thickness was significantly reduced at 1 day, 1 week, 1 month, and 3 months with ortho-K (*P* < 0.05). Paracentral (2 mm~5 mm annular ring) epithelial thickness was also significantly reduced at superior temporal, inferior temporal, temporal, and inferior locations after ortho-K (*P* < 0.05), while midperipheral (5 mm~6 mm annular ring) epithelial thickness was greater post- than pre-ortho-K at superior, superior temporal, inferior temporal, inferior, and inferior nasal locations (*P* < 0.05). In other zones, superior, superior nasal, nasal, and inferior nasal in paracentral annular ring and temporal and superior nasal in midperipheral ring, epithelial thickness underwent no significant change. Ortho-K lens wear caused the central corneal epitheliums to thin. The temporal half zones become thinner in paracentral zones and thicker in midperipheral zones.

## 1. Introduction

Orthokeratology, also referred as ortho-K or Ok, involves reverse-geometry design breathable and rigid contact lenses. Overnight ortho-K lens wearing results in reduced central cornea thickness temporally, reduced refractive error, and 20/20 or better unaided vision during the subsequent daytime with continuing wearing. Cho and Cheung [1] confirmed that ortho-K had the effect of slowing axial length progression and controlling myopia development in myopic children via myopic defocus. The central corneal curvature is flattened corresponding to relatively flat fitting base curve of ortho-K lens. And midperipheral cornea is steepened corresponding to relatively steep fitting reverse curve of ortho-K lens. Data display that central refractive power decreases and peripheral refractive power increases. Corneal topography shows a classic bull's eye because central cornea flattens and midperipheral cornea steepens after a well-centered ortho-K lens wear. The present study was designed to retrospectively research corneal epithelial changes post-ortho-K lens wear, which helps to explain corneal morphological changes.

Previous studies have reported central epithelial thinning and midperipheral epithelial thickening, measured by a modified optical pachymeter after 90 days of ortho-K lens wear [2]. Tao et al. measured the topographic thickness of the cornea and epithelium using spectral-domain OCT with ultrahigh resolution (~3 *μ*m) and concluded that the corneal epithelial thickness between the nasal side and temporal side had no significant differences [3]. In a further study, Du et al. used the same OCT as Tao's to measure the corneal epithelial thickness M at 10:00 AM and again at 6 and 8 hours later and confirm thinning of the superior midperipheral epithelium in the vertical meridian and thickening of the temporal and nasal midperipheries in the horizontal meridian [4]. Kim confirmed thinning of the central corneal epithelium and thickening of the midperipheral epithelium following ortho-K lens wear [5]. Zhang et al. [6] assessed the epithelial thickness in corneal zones before and after ortho-K lens wear using the RTVue OCT (Optovue Inc., Fremont, CA, United States) and found insignificant change in paracentral (2–5 mm) and midperipheral (5–6 mm) annular ring corneal epithelial thickness. There is a lack of consensus to date on corneal epithelium redistribution after ortho-K lenses wear. Swarbrick et al. [7] found that the epithelium measured using OCT was significantly thinner in ortho-K wearers than in nonlens wearers, not evolving paracentral and peripheral corneal epithelium. Interventions to control myopia take effect over time, so it is important to understand corneal changes that occur in prolonged ortho-K lens wear. The present study is the first to investigate central and zonal corneal epithelial changes over a period of up to three months of ortho-K lens wear.

## 2. Materials and Methods

### 2.1. Participants

Records of 78 children (146 eyes) aged between 8 and 13 years were reviewed in this study. The children had worn ortho-K lenses for 8 to 10 hours per night. The inclusion criteria were a spherical refractive error of less than 5.00 diopters (D) with astigmatism of 1.50 D or less, best-corrected distance visual acuity of 20/20 or better before treatment. And ortho-K lens centered or decentered by less than 1.0 mm radially determined by slit lamp examination. The exclusion criterion was any current ocular or systemic disease. All subjects were treated according to the tenets of the Declaration of Helsinki. A consent form was signed by the parent of each child.

### 2.2. Ortho-K Lens Fitting

Ortho-K lenses from Paragon CRT (Paragon Vision Sciences, Mesa, AZ, United States) were used for all subjects. Lens fitting was evaluated using fluorescein. The typical bull's eye pattern in the corneal topography confirmed an adequate fit after an overnight trial. At each visit after the beginning of ortho-K lens wear, a complete contact lens follow-up examination was performed, including slit lamp examination, visual acuity, corneal topography (Medmont, Arlington, WA, United States), and OCT to investigate the response to ortho-K lens wear.

### 2.3. Measurement of Corneal Epithelial Thickness

The corneal epithelial thickness data were obtained at baseline examination and during the follow-up visits. Unaided distance visual acuity of 20/20 or better and satisfactory topographic change were achieved in all patients and at all visits. We scanned the cornea in eight meridians using a Pachymetry+Cpwr scan over a 6 mm diameter zone centered at the corneal apex using the RTVue OCT system with a corneal anterior module set at a wavelength of 830 nm. The corneal epithelial thickness maps were divided into a total of 17 sectors using an automatic algorithm, including one central 2 mm diameter zone, eight paracentral zones within an annulus between 2 and 5 mm diameters, and eight midperipheral annuli between the 5 and 6 mm diameter ring zones. The mean measurements at the central zone and in each of eight measurements in the paracentral and midperipheral zones were analyzed statistically. All of the OCT images were performed by the same examiners. OCT was conducted after one day, one week, one month, and three months of ortho-K wear in 42, 40, 60, and 34 eyes, respectively (Table 1).

### 2.4. Data Analysis

SPSS version 21.0 for Windows (IBM Corp., Armonk, NY, United States) was applied to analyze statistically. Corneal epithelial thickness values were compared using the ANOVA test before and after OK lens wear. Data were recorded according to follow-up time (baseline, 1 day, 1 week, 1 month, and 3 months) to monitor the difference in refractive error and corneal thickness. One-way analysis of variance (ANOVA) was performed to compare differences among these 5 subgroups. All statistical significance was defined as *P* < 0.05.

## 3. Results

The study included 146 eyes of 30 male and 48 female subjects successfully treated with ortho-K lenses to control myopia progression. The mean age of the subjects was 10.2 ± 1.67 years (range: 7 to 14). Spherical equivalent refractive error reduced after treatment, as summarized in Table 1.

OCT was carried out after one day, one week, one month, and three months of ortho-K lens wear in 42 eyes, 40 eyes, 60 eyes, and 34 eyes, respectively. Not each subject attend all the routine aftercare visit due to personal reasons. At each of these time points, the flat and steep keratometry (*k*) readings were significantly reduced compared with pre-ortho-K readings. Compared with the one-day time point, flat and steep *k* readings were significantly reduced at one week, one month, and three months. However, the *k* readings at one week, one month, and three months were statistically similar (Table 2).

Flat *k* and steep *k* decreased at different time points significantly post-Ok compared with pre-Ok. Compared with 1-day post-Ok, flat *k* and steep *k* also decreased significantly at 1 week, 1 month, and 3 months post-Ok. Compared with 1-week post-Ok, flat *k* and steep *k* at 1 month and 3 months decreased not significantly. Flat *k* and steep *k* have no difference between 1-month and 3-month curvature.

### 3.1. Corneal Epithelial Thickness Changes

Compared with pre-ortho-K thickness, the central corneal epithelial thickness decreased significantly at each of the four time points (*P* < 0.05) (Table 3). Paracentral corneal thickness in the temporal zone was significantly decreased at day one; in the superior temporal, temporal, inferior temporal, and inferior zones at one week; in the temporal, inferior temporal, and inferior zones at one month; and in the superior temporal, temporal, and inferior temporal zones at three months (*P* < 0.05) (Table 3). In contrast, the midperipheral epithelial thickness was increased compared with pre-ortho-K levels in the inferior zone at one week; in the superior, superior temporal, and inferior temporal zones at one month; and in the inferior and inferior nasal zones at three months (*P* < 0.05) with no significant change in other zones at each of these time points (Table 3).

**Figure 1:**
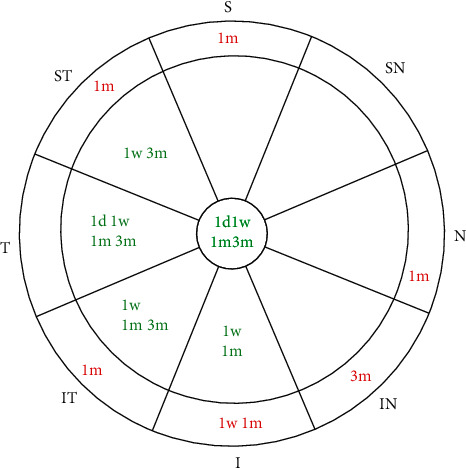
Corneal epithelium thickness changes after overnight ortho-K wearing. Green characters mean thickness decreases; red characters mean thickness increases.

There was a trend that overnight ortho-K remains stable at temporal direction. It was suggested that corneal epithelial redistribution occurred in the central and paracentral temporal, superior and inferior temporal, and inferior zones with migration to midperipheral temporal half-sectors within 6 mm zones surrounding the corneal apex, while the nasal zones did not change statistically (Figure 1).

## 4. Discussions

Designed with four to five curves, modern ortho-K lenses as a reverse-geometry contact lens have an enhanced centration and predictability of myopia control [8]. Ortho-K lenses are made of breathable materials, which are safe for overnight wear and allow subsequent daytime visual acuity to reach 20/20 or better. Orthokeratology has therefore become a common treatment to control myopia, arresting or slowing myopia progression [9–13] based on myopic defocus theory [14, 15]. The ortho-K lens central base curve is flatter than the cornea, and pressure is placed on the cornea from the lens eyelids and tear fluid. The reverse curve is steeper than the cornea providing space for central corneal epithelial migration. Corneal epithelial thickening in the reverse curve zones converts peripheral retinal hyperopic defocus into myopic defocus which may stop or slow axial length progression [14, 15].

Kim et al. [5] showed corneal epithelial thinning centrally and thickening in the midperiphery. The 5~6 mm annular ring showed no significant thickening compared to baseline values. Changes in epithelial thickness showed a nonuniform pattern with more thinning of the temporal and inferior zones compared with the nasal and superior zones of the paracentral region, with further thickening in the nasal zone compared with the temporal zone in the midperiphery [5]. In the present study, all of the thinning and thickening occurred at temporal half-zones in the 2~5 and 5~6 mm annuli. Thinning of the temporal 2~5 mm annular ring resulted in thickening of the temporal 5~6 mm annulus due to epithelial migration from the paracentral region to the midperiphery.

Morphological analysis performed using a confocal microscope showed significant structural changes particularly in the central epithelium after 15 days, one month, and one year after ortho-K wear [16, 17]. Our study confirmed that central corneal epitheliums were stably thinning till 3 months after ortho-K lens wear.

Wang et al. found thinning of the central epithelium and thickening in the midperiphery [18]. Several other studies have also found central corneal epithelial thinning [5–7, 19, 20] and flattening, with corneal curvature and myopia reduced and unaided visual acuity at 20/20 or better. In contrast with these findings, the present study found that thinning of the paracentral epithelium and thickening in the midperiphery happened at temporal half-meridian.

Zhang et al. [6] commented central epithelial thinning but no change in thickness at the 2–5 mm and 5–6 mm annular zones over time. The thickness at each time point after lens wear was significant compared with the thickness before lens wear. The present study demonstrated that epithelial thinning at the central 2 mm zone postlens wear reached the thinnest peak at one week post-ortho-K. The epithelium concordant with reverse curve area thickened after one day of lens wear and became stable at one week.

The reverse geometry of ortho-K lenses results in corneal epithelial morphological changes and redistribution. The base curve diameter of the lenses used in the present study is 6 mm. OCT images showed epithelial thinning in the central 2 mm, thinning in some paracentral zones in an annulus 2~5 mm from the corneal apex, and thickening in zones of the midperipheral annulus 5~6 mm from the apex. OCT measures the corneal central 6 mm zones and 5~6 mm annular ring area coincidence with the transition area from ortho-K lens' base curve to reverse curve. Additionally, 5~6 mm temporal half-annular area thickening is the anatomy basic of forming red myopic defocus ring displayed on the tangential corneal topography. Myopic defocus is a central theory in the control of myopia, and the present study provides a morphological basis for this theory.

The current research showed that thickness of the corneal epithelium was stable after one week of ortho-K lens wear in the 2 mm~5 mm paracentral annular ring and by one month in the 5-6 mm midperipheral annulus. Paracentral superior temporal, temporal, inferior temporal, and inferior corneal epithelium thinned significantly while other sectors thickened insignificantly. In the midperiphery, significant thickening was found in the superior, superior temporal, inferior temporal, inferior, and inferior nasal zones while changes in the nasal, temporal, and superior nasal zones were insignificant. As explained earlier, the findings are not in complete agreement with previous findings in this field.

The changes in corneal epithelial thickness all occurred in the temporal quadrants, both superior and inferior. It is known that human cornea is usually prolate ellipse and the nasal and temporal meridian eccentricity values are different [21, 22]. The cornea becomes flattening from center to periphery [23], and the flattening degree along the nasal meridians is greater than that along the temporal meridians from the center to the periphery [24]. After two weeks of ortho-K wearing, in the central circular zone, the temporal quadrant curvature is significantly flattened, and in the paracentral annular zone, the temporal curvature significantly steepened [25]. The current study included ortho-K lenses centered or decentered by no more than 1 mm as determined by slit lamp microscopy, yet all of the epithelial changes after ortho-K occurred in the temporal quadrants mainly. These results suggest that ortho-K had a trend to be stable at temporal quadrant not central zone and not nasal sector although nasal meridian becomes flatter than temporal one after ortho-K, which needs further researches. There is a limitation that the study is a retrospective one, which meant that some subjects were not followed up regularly and data collected were missed partly. Caution also needs to be taken; the current research subjects are children while the cornea shape during ortho-K may vary among adults or the elderly.

## Figures and Tables

**Table 1:** Eye number at different follow-up time points.

Follow-up time	1 day	1 week	1 month	3 months
Eye number	42	40	60	34

**Table 2:** Corneal curvature changes pre- and post-Ok lens wear.

	Pre-Ok	1 day post-Ok	1 week post-Ok	1 month post-Ok	3 months post-Ok
Steep *k*	43.86 ± 1.41	42.47 ± 1.60	41.43 ± 1.56	41.25 ± 1.56	41.22 ± 1.38
Flat *k*	42.74 ± 1.26	41.48 ± 1.44	40.59 ± 1.53	40.42 ± 1.63	40.46 ± 1.47

**Table 3:** Corneal epithelium thickness changes (average differences = thickness pre‐Ok − thickness post‐Ok).

	C	M S	M ST	M T	M IT	M I	M IN	M N	M SN	P S	P ST	P T	P IT	P I	P IN	P N	P SN
1 d	4.69^∗^	-0.27	0.99	2.23^∗^	1.83	0.96	-0.12	-0.61	-0.69	-0.72	-1.10	-0.32	-1.30	-1.33	-1.43	-0.10	-0.48
1 w	9.22^∗^	0.12	2.70^∗^	5.00^∗^	4.64^∗^	2.56^∗^	0.49	-0.46	-1.11	-2.30	-2.20	-0.53	-1.38	-3.10^∗^	-3.03	-1.65	-2.04
1 m	8.42^∗^	-1.44	1.94	4.50^∗^	3.97^∗^	2.13^∗^	0.09	-1.13	-1.56	-3.14^∗^	-2.86^∗^	-2.51	-4.18^∗^	-4.39^∗^	-3.04^∗^	-2.13	-2.31
3 m	7.41^∗^	-0.27	3.32^∗^	5.49^∗^	4.45^∗^	1.72	-0.55	-1.04	-1.63	-1.20	-0.59	0.89	-0.55	-3.86^∗^	-3.30^∗^	-1.32	-0.26

S = superior; ST = superior temporal; T = temporal; IT = inferior temporal; I = inferior; IN = inferior nasal; N = nasal; SN = superior nasal; C = central; M = midperipheral; P = peripheral. ^∗^*P* < 0.05.

## Data Availability

The data used to support the findings of this study are included within the article.
